# Role of NG2 expressing cells in addiction: a new approach for an old problem

**DOI:** 10.3389/fphar.2014.00279

**Published:** 2014-12-19

**Authors:** Sucharita S. Somkuwar, Miranda C. Staples, Melissa H. Galinato, McKenzie J. Fannon, Chitra D. Mandyam

**Affiliations:** Committee on the Neurobiology of Addictive Disorders, The Scripps Research InstituteLa Jolla, CA, USA

**Keywords:** prefrontal cortex, amygdala, hippocampus, progenitors, NG2, BrdU

## Abstract

Neuron-glial antigen 2 (NG2) is a proteoglycan expressed predominantly in oligodendrocyte progenitor cells (OPCs). NG2-expressing OPCs (NG2-OPCs) are self-renewing cells that are widely distributed in the gray and white matter areas of the central nervous system. NG2-OPCs can mature into premyelinating oligodendrocytes and myelinating oligodendroglia which serve as the primary source of myelin in the brain. This review characterizes NG2-OPCs in brain structure and function, conceptualizes the role of NG2-OPCs in brain regions associated with negative reinforcement and relapse to drug seeking and discusses how NG2-OPCs are regulated by neuromodulators linked to motivational withdrawal. We hope to provide the readers with an overview of the role of NG2-OPCs in brain structure and function in the context of negative affect state in substance abuse disorders and to integrate our current understanding of the physiological significance of the NG2-OPCs in the adult brain.

## INTRODUCTION

For centuries, the cellular composition of the healthy, intact mammalian brain was thought to be maintained in a static state, in the absence of cellular turnover. The relatively recent discovery of neural stem cells in all mammalian species, including humans, has forced researchers to adapt their understanding of the basal cortical structure and function under physiologic and pathologic conditions within the framework of this novel cellular phenomenon (Eriksson et al., 1998; Curtis et al., 2007). Nearly every major disorder which perturbs typical functioning of the brain, including addiction to illicit drugs, has been shown to influence or be influenced by rates of neural stem cell proliferation, differentiation, and/or survival (Canales, 2010; Mandyam and Koob, 2012; Drew et al., 2013). While much focus has centered on the role of generation of new neurons, or neurogenesis, it is critical also to evaluate the role of generation of new glia, or gliogenesis in typical and atypical cortical function. This review aims to highlight the role of a particular glial cell type, the neuron-glial 2 (NG2) cell in the adult mammalian cortex and speculate on the potential role of this specialized cell type in addiction to drugs of abuse.

## CHARACTERIZATION OF NG2 CELLS IN THE ADULT MAMMALIAN BRAIN

### NG2-GLIA: WHAT’S IN A NAME?

Glia in the brain are mainly classified into three subtypes: astroglia, microglia, and oligodendroglia. A fourth kind of proliferative glial cells termed NG2 cells that express their namesake characteristic marker, chondroitin sulfate proteoglycan, are found to be widely distributed throughout the gray and white matter of the adult rodent brain (Dawson et al., 2000, 2003; Levine et al., 2001). The notion that NG2 cells are oligodendrocyte progenitor cells (OPCs) was confirmed with *in vitro* studies demonstrating that NG2 labeled cells differentiated into oligodendrocytes in a differentiating culture preparation (Stallcup and Beasley, 1987); thus, these NG2 cells have been referred to as NG2-OPCs. Other names have been suggested for the NG2 cells, such as polydendrocytes to describe their multiple projections (Nishiyama et al., 2002), and synantocytes to describe their contact with neurons and astroglia (Butt et al., 2002). An in depth review on the biology and function of NG2 cells has been published elsewhere (Hill and Nishiyama, 2014; Tomassy and Fossati, 2014) and the current review will briefly discuss the phenotypic fate of NG2 cells and their role in the mammalian brain in the context of addictive disorders.

### PHENOTYPIC FATE OF NG2-GLIA

Following the initial identification of NG2 cells, one line of investigation pursued the phenotypic fate of these unique cells. NG2 cells isolated by immunopanning for A2B5 (an antibody that tags the ganglioside moiety expressed in pre-oligodendrocytes) revealed that NG2 cells *in vitro* mature into A2B5+ pre-oligodendrocytes (Abney et al., 1983; Baracskay et al., 2007), confirming that NG2 cells are directed into an oligodendrocyte phenotype. *Ex vivo* studies demonstrate that NG2 cells express two distinct markers of early oligodendroglial lineage, namely, platelet-derived growth factor α receptor (PDGFαR) and O-antigen 4 (O4), further supporting that NG2-cells are directed into an oligodendrocyte lineage in the adult brain (Reynolds and Hardy, 1997; Nishiyama et al., 1999; Dawson et al., 2003). Additional support for the differentiation of NG2-cells into oligodendrocyte phenotype comes from various genetic models and such studies confirm the direction of NG2 cells into oligodendrocytes and premyelinating oligodendrocytes *in vivo*. For example, using the NG2-CreERT2 transgenic line, it has been recently demonstrated that a large proportion of NG2 cells in various regions of the brain express oligodendroglial markers Olig2, Sox10 and adenomatous polyposis coli CC1 at various stages of development; embryonic brain [(E)17.5], postnatal brain (P3-P4), adolescent brain (P30-P34) and aged brain [10 month old; (Huang et al., 2014)]. Taken together, it is evident that NG2 cells are capable of generating oligodendrocytes all through the life-span of rodents.

Beyond NG2 cells developing into oligodendrocytes, several studies suggest that NG2 cells are mulitpotent cells that are capable of generating neuronal progenitor cells in addition to oligodendrocytes. For example, 50% of glioblastoma cells isolated from human subjects were co-labeled with NG2 and nestin, a marker expressed by neuronal progenitors (Svendsen et al., 2011). The *ex vivo* evidence is further supported by *in vivo* findings, where a significant proportion of NG2 cells expressed GFP in the transgenic nestin-GFP reporter mice (Ehninger et al., 2011). Furthermore, using transgenic lines Plp-CreERT2 and PDGFRα-CreERT2, postnatal NG2 cells were found to generate new neurons in the piriform cortex, albeit significantly lower in number when compared with oligodendrocytes (Doerflinger et al., 2003; Rivers et al., 2008; Guo et al., 2009). Taken together, these findings suggest that a small proportion of NG2 cells may have the capacity to generate neural progenitor cells and neurons in the adult brain.

While there is consistent evidence that small populations of NG2 cells can develop into neurons during adulthood, controversy remains over whether NG2 cells can similarly generate neurons during development. For example, using transgenic lines NG2-Cre and CreER BAC transgenic mice, it was demonstrated that embryonic NG2 cells did not mediate neurogenesis (Zhu et al., 2008b, 2011). These discrepancies in the neuronal phenotype of NG2 cells may be attributable either to variability in cell-specific expression of transgene due to non-homologous recombination strategies or to the developmental profile of NG2 cells in the central nervous system (Nishiyama et al., 2009, Richardson et al., 2011). To this end, a recent study attempted to overcome one limitation by using NG2-CreERT2 transgenic line using homologous recombination, thus enabling the transgene to be under the regulatory control of the endogenous regulators of the NG2 locus (Huang et al., 2014). In this study, inducing Cre activity in NG2 cells in the second postnatal week (P14) resulted in reporter gene colocalizing with neuronal markers such as NeuN and Tuj1 in the ventral cortex, suggesting that NG2 cells can differentiate into neurons in this brain region. Interestingly, inducing Cre activity during young adulthood (P30), did not reveal colocalization with neuronal markers in the previously demonstrated ventral cortex or regions of established adult neurogenesis (the hippocampal dentate gyrus and subventricular zone). Instead, the reporter-positive cells in the non-neurogenic regions in the adult brain (ventral cerebral cortex and hippocampal CA3 regions) were found to co-express neuronal markers NeuN and HuC/D. Given that NG2 cells generated adult born neurons in the non-neurogenic regions in the adult brain demonstrated morphology of interneurons and normal electrophysiological properties, it is tempting to speculate that NG2 cells indeed have neurogenic potential, but to a limited capacity and with brain region specificity compared with their more prolific oligodendrocyte potential (Huang et al., 2014). Alternatively, the NG2 cells with neurogenic potential may be a different population compared with the NG2 cells that differentiate into oligodendrocytes, and future studies are needed to confirm the multipotency of the NG2 cell population.

With respect to NG2 cells generating astroglial cells and microglial cells, there is evidence to both support and to reject the supposition. For example, the *in vitro* finding that NG2 cells differentiate into type-2 astrocyte led to the conceptualization of oligodendrocyte-type-2 astrocyte (O-2A) progenitor cells; this was, however, considered to be an *in vitro* artifact based on *ex vivo* and *in vivo* findings (Raff et al., 1983, Espinosa de los Monteros et al., 1993, Nishiyama et al., 2009). *Ex vivo* studies show that there is little or no evidence for co-labeling of astroglial specific markers [glial fibrillary acidic protein (GFAP)] and microglial specific markers OX-42 with NG2, however, the NG2 cells were reported in close apposition with astrocytes and microglia creating points of apparent overlap (Dawson et al., 2003, Mandyam et al., 2007). *In vivo* studies using transgenic mice have also been equivocal about the astroglial lineage of NG2 cells. Studies using the NG2-Cre and CreER BAC transgenic mice revealed that 40% of the protoplasmic astrocytes in the gray matter were generated from NG2 cells. In contrast, the radial glial cells in the gray matter and all the astroglial cells in the white matter were shown to arise from a non-NG2 lineage (Zhu et al., 2008a,b, 2011). Furthermore, in the Plp-CreERT2 transgenic mice, postnatal NG2 cells were found to generate astroglia in the ventral gray matter (Doerflinger et al., 2003; Guo et al., 2009). Additionally, using NG2-CreERT2 mice, NG2-CreER BAC transgenic lines as well as PDGFRα-CreER PAC and PDGFRα-CreERT2 BAC transgenic lines, no evidence was found to suggest that NG2 cells in mice (age ranging from P8 to adulthood) were capable of generating new astroglia (Rivers et al., 2008; Kang et al., 2010; Huang et al., 2014). Taken together, it appears that the astrogenic potential of NG2 cells may be restricted to certain brain regions and certain developmental periods; therefore more work is needed to confirm the astrogenic potential of NG2 cells in the adult brain.

From the evidence presented, it is clear that NG2-glia have the potential to develop into both neurons and astrocytes in adult animals, but develop primarily into oligodendrocytes; therefore, NG2 cells with oligodendrocyte potential will be the developmental lineage of focus for the remainder of the review and these cells will henceforth be called NG2-OPCs.

## ROLE OF NG2-OPCs IN THE ADULT MAMMALIAN BRAIN

Beyond the phenotypic fate of NG2-OPCs, it is critical to understand the role of NG2-OPCs in typical cortical function. While neurons are thought to be the most prominent postmitotic cells in the brain that are uniquely capable of generating action potentials as a means of communicating with other cell types and maintaining plasticity, this notion has been challenged by studies showing that NG2-OPCs exhibit several neuron-like properties. For example, NG2-OPCs juxtapose with pre- and post-synaptic neurons, and share direct synaptic connections with glutamatergic neurons, suggesting a potentially significant contribution to ongoing brain plasticity (Ong and Levine, 1999; Bergles et al., 2000; Paukert and Bergles, 2006). NG2-OPCs express glutamatergic α-amino-3-hydroxy-5-methyl-4-isoxazolepropionic acid (AMPA) receptors and regulate extrasynaptic glutamate, suggesting that these cells may be involved in glutamatergic signaling (Dawson et al., 2003; Stegmuller et al., 2003; Karram et al., 2005; Paukert and Bergles, 2006). Even more interesting was the finding that some (but not all) NG2 cells, in both the gray and white matter regions of the brain, are capable of generating action potentials (Chittajallu et al., 2004; Ge et al., 2006; Karadottir et al., 2008) and display activity-dependent cellular plasticity (Ge et al., 2006). Therefore, it can be hypothesized that there are two distinct populations of NG2-glia based on the expression of (or lack of expression of) voltage-gated Na^+^ channels and formation of glutamatergic synapses; alternatively, expression of voltage-gated Na^+^ channels and the associated cellular characteristics are typical of a few but not all stages of maturation of NG2-OPCs. Another open–ended question is whether the two populations of NG2-OPCs, if present, are functionally distinct, or have distinct capacities for differentiating into oligodendrocytes. It is tempting to speculate that the NG2-glia expressing voltage-gated Na^+^ channels are either terminally differentiated or are designated to a neuronal fate, and hence are not capable of generating oligodendrocytes *in vivo*. Recent studies using NG2-reporter transgenic mice have empirically evaluated these questions (De Biase et al., 2010). The study demonstrated that NG2-OPCs received synaptic inputs from glutamatergic neurons during early postnatal development. However, all NG2-OPCs did not respond similarly to the glutamatergic inputs, where some NG2-OPCs exhibited depolarization but none generated action potential. Furthermore, differentiation of NG2-OPCs into oligodendrocytes resulted in downregulation of both voltage-gated Na^+^ channels and glutamatergic receptors (De Biase et al., 2010). These findings suggest that NG2-OPCs have distinct physiological roles during development and during adulthood. Thus, these studies confirm that NG2-OPCs can contribute significantly to ongoing synaptic plasticity, predominantly during the critical stages of neural development.

Neuron-glial antigen 2 is the hallmark protein of the NG2-OPCs and is expressed in the cell body as well as the processes radiating from the soma (Dawson et al., 2003). Several physiological roles have been suggested for the NG2-OPCs. For example, the proteoglycan shares structural features with neural cell adhesion molecules, and mediates the physical and synaptic connections between NG2 cell processes and the cell membrane of pre- and post-synaptic neurons (Bergles et al., 2000; Stegmuller et al., 2003). NG2-OPCs function as precursors to oligodendrocytes and closely interact with axons in various brain regions including the corpus callosum, cortex, and hippocampus [further reviewed in section **role of NG2-OPCs in addiction;** (Kukley et al., 2007; Ziskin et al., 2007; Karadottir et al., 2008; Ge et al., 2009; Etxeberria et al., 2010)]. Electrophysiological studies revealed glutamatergic synapses transmitting AMPA receptor-mediated currents between axons and NG2-OPCs, indicating functional synapses (Ziskin et al., 2007; Etxeberria et al., 2010). Furthermore, it has been demonstrated that the NG2-axon synapses could help to promote growth and myelination after nerve injury (Yang et al., 2006). Such studies allow us to speculate that the NG2-OPC – axon synapses may convey or receive information about the microenvironment, which could assist with triggering trophic signaling pathways that can aid in proliferation and differentiation of NG2-OPCs. Nevertheless, it can be hypothesized that the NG2-OPC – axon synapses allow for quick responses to injury, particularly those requiring remyelination processes.

Indeed, NG2-OPCs respond to axon injury by increasing their capacity to proliferate and differentiate into myelinating oligodendrocytes (Ong and Levine, 1999; Chida et al., 2011; Xiong et al., 2013). Such responses have been demonstrated in multiple types of demyelinating models of axon injury including hypothermia (Xiong et al., 2013), kainate (Ong and Levine, 1999), ischemia (Chida et al., 2011), and cuprizone (Kumar et al., 2007), where NG2-OPC density was enhanced at the site of injury concurrently with increased expression of myelin basic protein (MBP; a marker for myelinating oligodendrocytes). Notably, the NG2-OPC response to demyelinating lesions has been associated with up regulation of the trophic factor brain-derived neurotrophic factor (BDNF; VonDran et al., 2011), suggesting a cell-intrinsic type mechanism. These results demonstrate a functional role of NG2-OPC up regulation in maintaining myelin plasticity in the adult brain (Patel et al., 2010; Chida et al., 2011; Xiong et al., 2013).

## DEVELOPMENTAL STAGES OF NG2-OPCs IN THE ADULT MAMMALIAN BRAIN

### PROLIFERATION OF NG2-OPCs

**FIGURE 1:**
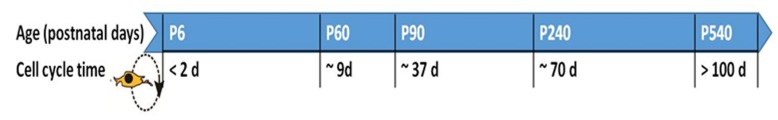
**Cell cycle time for NG2-OPCs in the rodent brain at different postnatal (P) ages.** The cell cycle has been found to slow down with age, and this effect has been reported in the cortex as well as corpus callosum (Psachoulia et al., 2009; Simon et al., 2011). Age in days post-partum; cell cycle time in days (d).

Neuron-glial antigen 2-OPCs are predominantly found in the corpus callosum and in the gray matter regions of the brain (Ong and Levine, 1999), where they continue to proliferate during adulthood. Experiments using mitotic markers of cellular proliferation such as 5-bromo-2′-deoxyuridine (BrdU) in mice have shown that the cell cycle dynamics of NG2-OPCs are affected by age (**Figure 1**). Cell cycle time increases with age, where NG2-OPCs cycle through one cell cycle of ~70 days beyond postnatal day (P)240 compared with a cell cycle length of 2 days at P6 (Psachoulia et al., 2009). These changes in cell cycle dynamics could support the decline in density of NG2-OPCs to approximately 75% of the initial cell mass in older animal subjects compared with newborn and young adults (He et al., 2009). These studies allow us to hypothesize that the cell cycle dynamics (such as length of time a cell spends in the cell cycle) of NG2-OPCs during development regulates the differentiation of the cell, such that reducing the length of the cell cycle assists with maintenance of the undifferentiated state (Lange et al., 2009; Salomoni and Calegari, 2010).

### DIFFERENTIATION OF NG2-OPCs

**FIGURE 2:**
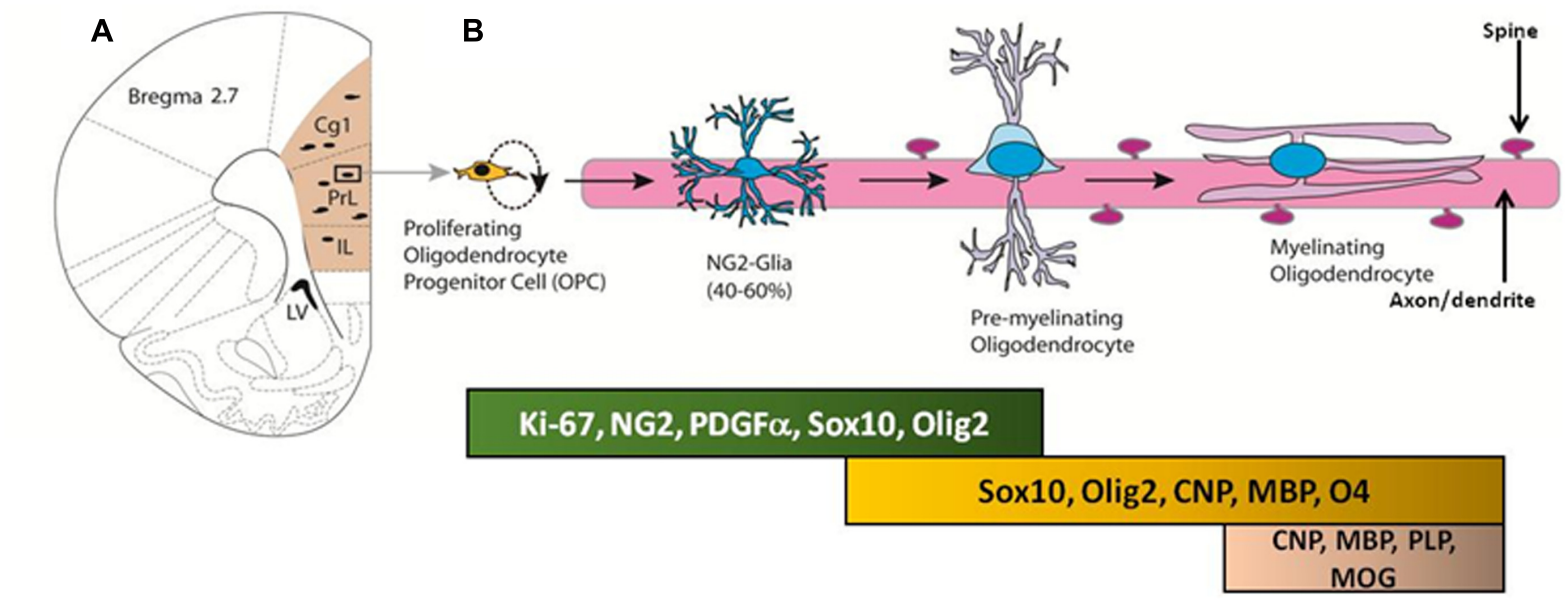
**(A)** Coronal section of the adult rat brain through the mPFC; the mPFC is shaded in peach. Proliferating cells in the mPFC differentiate into oligodendrocyte progenitor cells (OPC) and premyelinating oligodendrocytes. **(B)** Developmental stages of oligodendrocyte progenitors in the mPFC; proteins and transcription factors that are expressed in distinct stages are indicated below.

Neuron-glial antigen 2-OPCs in the adult brain primarily differentiate into premyelinating oligodendrocytes and myelinating oligodendrocytes, which produce myelin to maintain white matter tracts (**Figure 2**; Dimou et al., 2008; Rivers et al., 2008; Zhu et al., 2008a,b, 2011; Geha et al., 2010; Kang et al., 2010; Clarke et al., 2012). The oligodendrocyte lineage of NG2 cells is dependent upon expression of basic helix-loop-helix transcription factors OLIG1 and OLIG2 (Zhu et al., 2012). For example, Zhu et al. (2012) used conditional knockout mice to cause constitutive and inducible deletions of *Olig2* specifically in NG2 cells to show that when *Olig2* is knocked down, NG2 cell fate switch to astrocytes rather than oligodendrocytes (Zhu et al., 2010). Such loss-of-function genetic studies confirm the importance of OLIG2 expression in directing the NG2-OPCs to a myelinating oligodendrocyte lineage. Recent research supports the generation of myelinating oligodendrocytes from the proliferating pool of NG2-OPCs during adulthood, and these cells eventually express markers associated with myelin, such as myelin basic protein (Rivers et al., 2008; Geha et al., 2010; Clarke et al., 2012). However, the functional significance of adult-generated myelinating oligodendrocytes remains to be explained. Below we will briefly discuss an important marker that is key to the process of differentiation of NG2-OPCs into myelinating oligodendrocytes.

#### OLIG1 and OLIG2

Oligodendrocyte transcription factor 1 and 2 (OLIG1 and OLIG2) are transcription factors that are predominantly expressed in OPCs and in myelinating oligodendrocytes (Takebayashi et al., 2000; Zhou et al., 2000). Structurally, these transcription factors dimerize at the basic helix-loop-helix region and bind to their DNA targets to regulate transcription of other genes associated with ongoing biological functions such as neurogenesis and reactive gliogenesis (Fancy et al., 2004; Buffo et al., 2005; Menn et al., 2006). Detailed descriptions of the structural and functional differences between OLIG1 and OLIG2 have been recently published elsewhere and the readers are referred to the review by Meijer et al. (2012). Briefly, OLIG1 regulates the expression of several genes involved in oligodendroglial maturation, including MBP, myelin oligodendrocytic glycoprotein, myelin proteolipid protein, and zinc finger protein 488 (Arnett et al., 2004; Xin et al., 2005; Wang et al., 2006; Guo et al., 2010). Additionally, OLIG2 has been found to play several critical roles in oligodendrocyte differentiation including enhancing the expression of Sox10 and Sip 1, proteins that enhance oligodendrogial activity and maturation of NG2-OPCs (Wang et al., 2006; Kuspert et al., 2011; Weng et al., 2012; Yu et al., 2013). However, OLIG2 also has been identified as a transcription repressor for several targets and consequently has been implicated in human glioma (Lee et al., 2005; Ligon et al., 2007; Mehta et al., 2011). For example, OLIG2 is also implicated in modulating the response of non-oligodendrocytic glial cells, such as in reactive gliogenesis (proliferation of astrocytes and microglia) following a demyelinating injury in the cortex (Arnett et al., 2004; Chen et al., 2008b). These findings are in apparent contradiction to studies that reveal an absence of lineage relationship between OLIG 1/2 expressing cells and astrocytes, as well as studies showing that NG2-OPCs are not progenitors of reactive astroglia (Dimou et al., 2008; Komitova et al., 2011). These contradictions may be explained by the transient, non-lineage-dependent expression pattern of OLIG2 in the reactive astroglia (Magnus et al., 2007; Zhao et al., 2009).

## ROLE OF NG2-OPCs IN ADDICTION

Addiction to illicit drugs has taken emotional and financial tolls on society, cutting across ages, races, ethnicities, and genders. Eventual dependence on any illicit drug increases mortality, morbidity, and economic costs. Despite the increase in the prevalence of addiction to illicit substances and the market for novel therapeutics, the research into understanding the neurobiological basis of addiction and relapse has progressed less well. Broadly defined, addiction is one of the many disorders that involves impulsivity and compulsivity (Heilig and Koob, 2007; Koob and Volkow, 2010), in which the impulsive phase involves the pleasurable effects of the drug and upon abstinence produces reward-induced craving and the compulsive phase develops after prolonged use of the drug when the individual seeks avoidance of the negative effects associated with drug withdrawal. The impulsive and compulsive phases of addiction can be characterized into three stages: (1) binge/prolonged intoxication, (2) withdrawal neutral/negative affect, and (3) preoccupation/anticipation (craving). The last stage of the addiction cycle describes a key element of relapse in humans and therefore defines addiction as a chronic relapsing disorder. Relapse to drug-seeking behavior is one of the least studied aspects of addiction, which has been a challenging area for neuroscientists.

Utilizing intravenous self-administration models of drug exposure, the research on addiction indicates dysregulation of the ‘hedonic set point’ and alteration in allostasis of the brain reward system which underlies the relapse to drug seeking and consequently addiction to the drug of abuse (Koob and Nestler, 1997; Koob and Le Moal, 2001, 2005). The relapse circuitry in adult mammalian brain is carved out based on multiple groundbreaking studies performed in rodent models of reinstatement (Shaham et al., 2003). The key brain regions implicated in the reinstatement of drug-seeking behavior include, but are not limited to, the prefrontal cortex (PFC), nucleus accumbens (NAc), bed nucleus of the stria terminalis (BNST), amygdala, hippocampus and the ventral tegmental area (VTA; Shaham et al., 2003; Koob and Volkow, 2010). The mesocorticolimbic dopamine system possesses neural connections from the VTA to the PFC and NAc and was crowned as the key circuit for reward and reinstatement of drug-seeking behaviors (Schultz, 1998; Horvitz, 2000; Robinson and Berridge, 2000; Comoli et al., 2003; Steketee, 2005). Most importantly, it is believed that the release of the neurotransmitters such as dopamine, glutamate, and corticotrophin-releasing factor in the key brain regions associated with relapse are essential for the behavioral outcomes of the drug (Wise, 1998; Di Chiara, 1999; Koob, 1999; Lu et al., 2003; Knackstedt and Kalivas, 2009). Furthermore, recent evidence supports the hypothesis that elevated anxiety, low mood, and increased sensitivity to stress (collectively labeled as negative affect) is the driving force behind the transition to addiction (George et al., 2014). Particularly interesting is the accumulating evidence that pathological neuroadaptations in the mPFC, extended amygdala and hippocampus may contribute to the negative affect state (Deschaux et al., 2012; George et al., 2012; Vendruscolo et al., 2012; Cohen et al., 2014). The following topics will characterize the NG2-OPCs in the brain regions associated with the negative affect state and hope to provide promising insights into translating the science of NG2-OPCs to future novel therapeutic approaches to target the relapse stage of the addiction cycle.

### CHARACTERIZATION OF NG2-OPCs IN THE PREFRONTAL CORTEX

The PFC regulates executive functions such as decision making, impulse control and working memory (Bechara et al., 1994, 2001). Decreased PFC function has been associated with addiction, such that preexisting impairments in PFC function (as seen in attention deficit hyperactivity disorder and schizophrenia) serve as predictors for enhanced vulnerability for addiction (Crews and Boettiger, 2009; de Wit, 2009; Groman et al., 2009; Wing et al., 2012). Furthermore, chronic use of addictive drugs has been shown to enhance functional deficits in the PFC, particularly those related to decision making and impulse control, as a mechanism to perpetuate the recurrent relapsing drug-addicted phenotype (Franklin et al., 2002; Johnson et al., 2008; Xiao et al., 2008; Crews and Boettiger, 2009; de Wit, 2009; Koob and Volkow, 2010).

**FIGURE 3:**
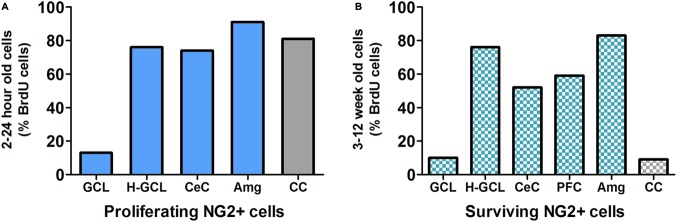
**(A)** NG2 positive (NG2+) cells are the predominant proliferating cell type in most brain regions except in the granule cell layer (GCL) of the hippocampus. Data derived from previous publications (Dawson et al., 2003; Wennstrom et al., 2004; Orre et al., 2009; Hattiangady and Shetty, 2010; Ehninger et al., 2011). **(B)** Survival/persistence of NG2+ cells is low in the GCL of the hippocampus. In contrast more than 50% of the surviving BrdU cells are NG2+ in the hippocampal non-GCL areas, cerebral cortex and prefrontal cortex (PFC) as well as the amygdala. Data derived from previous publications (Wennstrom et al., 2004; Komitova et al., 2006; Czeh et al., 2007; Mandyam et al., 2007; Orre et al., 2009; Hattiangady and Shetty, 2010; Ehninger et al., 2011). GCL, granule cell layer; H-GCL, hippocampus without GCL; CeC, cerebral cortex; PFC, prefrontal cortex; Amg, amygdala; CC, corpus callosum.

Studies have shown that within the mammalian cortex there is an abundant NG2 cell population that forms an evenly distributed and dense network (**Figure 3**; Dawson et al., 2003; Girolamo et al., 2010), however, the explicit role of NG2-OPCs in the mammalian cortex is still unclear. Recent studies in rodents have been directed at determining the role of these unique cells. While functional implications of NG2-OPCs are of critical interest, most recent studies of such cells in the cortex, and more specifically the PFC, are targeted at understanding proliferation and differentiation of NG2-OPCs.

Due to the important role the PFC plays in mood disorders such as depression, research with regard to this clinical condition has added information to further the understanding of this issue. Studies focusing on electroconvulsive seizure (ECS) treatment have explored phenotypic analysis and proliferation of cells in the PFC. Studies showed that in addition to an increase in proliferation of cells following ECS treatment, there is a larger proportion of NG2-OPCs when compared with other cell types such as astrocytes, neurons, or endothelial cells. While some studies show only a modest increase in the number of NG2-OPCs, others demonstrate that 40–50% of newly born cells are co-labeled as NG2 positive cells (Madsen et al., 2005; Czeh et al., 2007; Ongur et al., 2007). Other cell types, such as endothelial cells, were detectable but at a much lower proportion than NG2-OPCs. Astrocytes and neurons were consistently absent from such phenotypic analysis in these ECS studies. Additional inquiry into the role of NG2-OPCs in the cerebral cortex has revealed that stab wound injuries to the cortex also produce a large proportion of NG2-OPCs in the injured area, in this case as much as five times as many NG2 cells when compared to non-injured controls (Simon et al., 2011). This evidence is very similar to the previous findings in the PFC region.

Depression and affective disorders are not the only realm where NG2 cells in the PFC play an important part. Drug abuse research considers the PFC function to be an important factor in the negative reinforcement associated with motivational withdrawal and research in this field has demonstrated increased levels of NG2-OPCs in the PFC when animals are exposed to intermittent exposure to powerful psychostimulants like methamphetamine (Mandyam and Koob, 2012). Chronic exposure to methamphetamine or to sedative-hypnotics, such as alcohol, proves to be too toxic for these NG2-OPCs to survive (Mandyam et al., 2007; Richardson et al., 2009; Kim et al., 2014). Therefore, these studies support the hypothesis that the glial disturbance observed with chronic drug exposure may serve as an indirect mechanism to promote neurotoxicity.

The only deviation with regards to cellular differentiation from previously discussed studies is discovered when examining the effect of other proliferation enhancing treatments such as physical activity. Animals were given voluntary access to running wheels and were examined postmortem to determine cellular phenotype in the PFC (Mandyam et al., 2007). In contrast to studies observing the effects of harmful processes on cell differentiation in the PFC, the wheel running data showed that newly born cells in the PFC are still mostly labeled as NG2-OPCs, but there were higher amounts of other kinds of cells observed such as neurons and astrocytes than have been seen in other studies. Therefore, there may be a relationship between the overall environment of the PFC and the capability of cells to differentiate into different cell types. Another study supports this hypothesis in the cerebral cortex by observing that when voluntary physical exercise was provided to animals, there was a reduced population of NG2-OPCs and increased levels of premature differentiation into oligodendrocytes (Simon et al., 2011).

Collectively, the data suggests that when there is an insult to the brain, say for example, by depression, stab wound or drug abuse, a large population of NG2-OPCs is needed to protect against these harmful effects and does not allow these cells to mature into different types of cells. Whereas if there is already a protective mechanism in place, like exercise, NG2-OPCs are still abundant in the PFC but allow for a wider range of maturation within the cell population. One last piece of compelling evidence was seen using *in vivo* recordings via cranial windows to observe NG2-OPCs. Following focal laser lesions in the cortex, NG2 cells were seen migrating to the injured sites and eventually surrounding the site and contributed to forming a glial scar (Hughes et al., 2013).

In summation, studies focusing on NG2 in the cortex and PFC have shown that NG2-OPCs are decreased along with the entire cell population in pathology. Additionally, NG2-OPCs are increased with treatments like pharmacological and physiological interventions. However, exercise supported the proliferation and differentiation into astrocytes and neurons. Taken together, this evidence provides support that NG2-OPCs in the PFC are involved in repair processes following insult.

### CHARACTERIZATION OF NG2-OPCs IN THE HIPPOCAMPUS

The hippocampus is involved in learning and memory, cognitive function as well as mood regulation (Li et al., 2009; Jun et al., 2012). While the dorsal hippocampus has been implicated in modulating declarative and contextual memory, the ventral hippocampus via its reciprocal connections with the amygdala has been implicated in regulation of mood; both of these systems undergo maladaptive changes following chronic exposure to drugs of abuse (White, 1996; Volkow et al., 2004; Koob and Volkow, 2010). Adult neurogenesis in the hippocampal subgranular zone is particularly sensitive to chronic drug induced disruption leading to aberrant adaptions in memory (Canales, 2007, 2010; Jun et al., 2012). Neuroadaptions in the hippocampus are particularly important as they contribute to enhanced sensitivity to context as well as stress induced relapse (Kilts et al., 2001; Crombag et al., 2008; Koob, 2008; Koob and Volkow, 2010; Belujon and Grace, 2011).

The existence of NG2-OPCs in the hippocampus is well established. In the normal adult, NG2-OPCs are distributed throughout all layers of the hippocampus (Levine and Nishiyama, 1996; Ong and Levine, 1999; Xu et al., 2014) with higher numbers in the stratum lucidum and dentate hilus (**Figure 3**; Bu et al., 2001). The functional role of NG2-OPCs in the hippocampus, however, remains to be found. Possible functions can be discussed based on what is known about NG2 cell development, NG2 cell interactions with other cell types, and NG2 cell reactions to changes in the hippocampus.

Neuron-glial antigen 2-OPCs are present and proliferate both during development and into adulthood. The density of NG2-OPCs in the adult hippocampus changes with age (Levine and Nishiyama, 1996; Chen et al., 2008a, Hughes et al., 2013). Initially, the number of NG2-OPCs increased from P0–P7, and then decreased when measured at P21, P50, and P450 as evidenced by NG2 immunostaining and NG2 protein level expression (Chen et al., 2008a). NG2 cell morphology also changes with age, with neonatal rats expressing NG2 in cells with ‘simpler’ morphology, and ‘complex’ morphology appearing by P7. Most of the NG2-OPCs in the adult hippocampus have a stellate morphology with several processes radiating from the soma (Ong and Levine, 1999; Chen et al., 2008a; Xu et al., 2014). NG2-OPCs are motile and extend processes to survey the surrounding environment. NG2-OPCs maintain homeostatic control of cell density through a balance of differentiation, proliferation, cell-death, and self-repulsion (Hughes et al., 2013).

The proliferation and differentiation of NG2-OPCs in the hippocampus has been described extensively [for review see (Richardson et al., 2011)]. NG2 cells are considered to be NG2-OPCs that eventually differentiate into myelinating oligodendrocytes (Butt et al., 1999; Nishiyama et al., 1999, 2009; Wigley et al., 2007; Kang et al., 2010); however, there are studies that investigated the multipotency of NG2 cells in the CNS (Kondo and Raff, 2000; Sypecka et al., 2009). There is evidence that hippocampal cell cultures provide a neuronal micro-environment that can induce NG2-derived neurogenesis (Sypecka et al., 2009). However, several fate-mapping studies in transgenic mice show that in the hippocampus, NG2-OPCs primarily develop into myelinating oligodendrocytes (Zhu et al., 2008a,b, 2011).

The spatial organization of NG2-OPCs led researchers to investigate communication between NG2-OPCs and other cell types. NG2-OPCs are in close contact with astrocytes and neurons (Nishiyama et al., 1999; Ong and Levine, 1999; Xu et al., 2014). There is now surmounting evidence of NG2-OPCs making synaptic contact with neurons [for review see (Karram et al., 2005)]. Neuron-NG2 cell synapses have been identified by electrophysiological techniques (Bergles et al., 2000; Lin and Bergles, 2004; Jabs et al., 2005; Ge et al., 2006; Kukley et al., 2008; Mangin et al., 2008). Kukley et al. (2010) used a NG2cre:Z/EG double-transgenic mouse line to identify developmental stages of NG2-OPCs and see at what stages they make synaptic contact with neurons in CA1 of hippocampus. Whole-cell patch clamp recordings revealed that synaptic input is restricted to NG2-OPCs, and is lost when they differentiate into premyelinating and myelinating oligodendrocytes (Kukley et al., 2010).

AMPA receptors that are calcium permeable have been found on hippocampal NG2-OPCs (Seifert and Steinhauser, 1995; Bergles et al., 2000; Lin and Bergles, 2002). NG2-OPCs can respond to neuronal stimulation and neurotransmitter release because they harbor *N*-methyl-D-aspartate (NMDA) receptors, AMPA receptors and γ-aminobutytic acid (GABA) receptor subtype A (GABAA; Bergles et al., 2000; Lin and Bergles, 2002, 2004; Chittajallu et al., 2004; Jabs et al., 2005; Karadottir et al., 2005; Salter and Fern, 2005; Ge et al., 2006; Paukert and Bergles, 2006; Kukley et al., 2007). Neuron-NG2 synapses can have activity-dependent changes analogous to long-term potentiation (LTP) at glutamatergic neuronal synapses. LTP expression in neuron-NG2 synapses is mediated by calcium-permeable AMPA receptors located on NG2-OPCs (Ge et al., 2006).

Connections between neurons and NG2-OPCs allow NG2 cells to be highly responsive to injuries in the hippocampus, inducing different morphological and proliferative changes over time (Nishiyama et al., 1999; Bu et al., 2001). After kainic acid-induced excitotoxic lesions in the hippocampus, NG2-OPCs showed two types of reactive changes: the early and persistent change 24 h to 3 months after lesion and the late, transient change 2 weeks after lesion (Bu et al., 2001). The first early change was characterized as an increase in NG2 immunoreactivity and an increase in processes extending from the cell body. NG2-OPCs during the late change had large round cell bodies, had short processes and they also expressed OX42 and ED1, markers for microglia/macrophages. There was also a corresponding change in the distribution of GFAP+ astrocytes in CA3. At 3 days post lesion, NG2 reactivity was high while GFAP cells were low, and at 2 weeks post lesion, NG2 reactivity was reduced while astrocytes filled in (Bu et al., 2001). Increases in NG2 reactivity is similar for multiple injury types, including inflammation (Nishiyama et al., 1997), viral infection (Levine et al., 1998), mechanical wound (Levine, 1994), ECSs (Jansson et al., 2009) and excitotoxic lesion (Ong and Levine, 1999). Similar changes in morphology and antigen expression in NG2-OPCs occurred in parallel with activation of microglia using a model of selective neurodegeneration in the mouse dentate gyrus with trimethyltin (Fiedorowicz et al., 2008). The monocyte properties of NG2-OPCs suggest that the NG2 cells may serve a function in phagocytosis.

The response of increasing NG2-OPC proliferation can be the first stages of a myelination response. Lesions of the entorhino-hippocampal perforant pathway induces formation of axonal sprouts, which recruits NG2-OPCs to divide and become oligodendrocytes as evidenced by NG2 cells and oligodendrocytes incorporating BrdU 9 weeks post lesion (Drojdahl et al., 2010). One way NG2-OPCs respond to the environment is through acid chemosensors, specifically the ASIC1a channel found in the cell membrane. Activation of these channels can induce membrane depolarization and Ca^2+^, which would serve as a quick response to injury following ischemia (Lin et al., 2010). It appears that NG2-OPCs in the hippocampus function to maintain glial homeostasis. Their responses to mechanical and cellular injury suggest that NG2-OPCs in the hippocampus may assist with maintaining hippocampal myelin plasticity.

### CHARACTERIZATION OF NG2-OPCs IN THE AMYGDALA

The amygdala is involved in modulating emotional memory and affective behavior, and is particularly important for fear learning and adverse reactions; these behavioral maladaptations in the amygdala are associated with negative reinforcement which triggers relapse to drug seeking (Grant et al., 1996; Maren, 1999; Blair et al., 2001; Kilts et al., 2001; Funk et al., 2006; Koob, 2008). Structural abnormalities in the amygdala are associated with the pathophysiology of addiction and several neurological disorders, including but not limited to depression, schizophrenia and temporal lobe epilepsy (Sheline et al., 1998; Mervaala et al., 2000; Wright et al., 2000; Drevets et al., 2002; Faber-Zuschratter et al., 2009; Chen et al., 2014). Several studies have uncovered neuronal aspects of amygdalar plasticity (for example synaptic plasticity and neurotropic mechanisms) in emotional behaviors [for review, (Maren, 2005)], however few studies have evaluated gliogenesis in amygdalar plasticity.

Amygdalar NG2 cells share several similarities with NG2-OPCs in the cortex and hippocampus. Approximately, 88–94% of the proliferating cells in the adult rodent amygdala were NG2-OPCs (**Figure 3**), and this pool of NG2 cells appear to exhibit limited differentiation over time (Wennstrom et al., 2004; Ehninger et al., 2011). These cells exhibit electrophysiological properties that were similar to the NG2-OPCs from corpus callosum (Ehninger et al., 2011). Similar to hippocampus and PFC, ECSs [established therapeutic strategy for depression (Clarke et al., 1989)], increased proliferation of NG2-OPCs (NG2+/BrdU+), increased expression of NG2 protein (Jansson et al., 2009), and subsequent differentiation into mature oligodendrocytes 3 weeks after cell division (RIP:oligodendrocyte marker and BrdU+) in the amygdala (Wennstrom et al., 2004; Jansson et al., 2009). Furthermore, oral administration of lithium chloride (established treatment for bipolar disorder) was reported to increase proliferation of NG2-OPCs in the amygdala (a similar response compared with the hippocampus and PFC), but enhanced proliferation was not associated with an increase in differentiation of NG2 cells into mature oligodendrocytes (Orre et al., 2009). However, physical activity via wheel running and environmental enrichment in rats did not alter the proliferation or the survival of newly born NG2-OPCs in the amygdala (an opposite response compared with the hippocampus and PFC), but decreased formation of new astroglia (S100β: astroglial marker and BrdU+) in the amygdala (Ehninger et al., 2011). The differences in responses to the wheel running stimulus could be due to variability in experimental design as the study in the amygdala used control rats that were not maintained under impoverished environmental conditions [for description impoverished environment, (Bardo et al., 1997)]. Taken together, research thus far suggests that amygdalar NG2-OPCs proliferation is a neuroplastic response restricted to certain pharmacological or mechanical type injuries.

In the above context, amygdalar gliosis and aberrant NG2 cell expression is associated with temporal lobe epilepsy (Faber-Zuschratter et al., 2009). Structural abnormalities in the amygdala are associated with the pathophysiology of mood disorders like depression (Sheline et al., 1998; Mervaala et al., 2000; Drevets et al., 2002) and of psychiatric disorders such as schizophrenia (Wright et al., 2000; Chen et al., 2014). In fact, postmortem studies suggest that schizophrenia patients exhibit decreased oligodendrocyte density in the amygdala compared with age-matched controls (Uranova et al., 2004; Williams et al., 2013). It appears that much more data is available in the amygdala to demonstrate that NG2 glial disturbance occurs in response to mood disorders and neurodegenerative diseases. However, further studies are required to uncover the role of NG2-OPCs in the amygdala in addiction and relapse to drug seeking.

### NEUROMODULATORS OF STRESS RESPONSES AFFECT NG2-OPCs

Addiction and eventual dependence to illicit drugs and alcohol induces attenuated (opposing) basal stress hormone levels [adrenocorticotropic hormone (ACTH) and corticosterone (CORT) an agonist at the glucocorticoid receptor (GR) and the mineralocorticoid receptor (MR)] compared with non-dependent subjects (enhanced stress hormone levels), and it has been demonstrated that the blunted stress response is a consequence of chronic drug or alcohol exposure (Zorrilla et al., 2001; Mandyam et al., 2008; Richardson et al., 2008). Importantly, the findings from the animal studies are consistent with clinical studies that link maladaptive hypothalamic-pituitary-adrenal (HPA) axis function with drug dependence and alcoholism, including a reduced ability to cope with stress and negative correlations between cortisol and craving and relapse in dependent subjects (Lovallo et al., 2000; Winhusen and Somoza, 2001; O’Malley et al., 2002; Nava et al., 2006). Although the precise mechanism underlying the attenuated stress response is unknown, several studies have implicated activation of pro-stress hormones [via enhanced expression of corticotrophin releasing factor (CRF) and altered expression of the CRF receptors] in the extended amygdala to contribute to the dysregulated stress system associated with dependence (Wand, 2005; Koob, 2008). Furthermore, enhanced GR levels in the extended amygdala during protracted abstinence has been demonstrated in dependent animals and such associated changes in GR system could play a mechanistic role in sensitivity to stress/reward and relapse associated with dependence (Vendruscolo et al., 2012). However, the functional significance of altered GR system in mediating blunted stress responses in drug dependence is unknown.

The few select studies that have attempted to ascertain a relationship between stress and NG2-OPCs have established a role for CORT in the proliferation, differentiation, and generation of myelin of these developmentally derived cells that appears to be dose and duration dependent (Chari, 2014). Oligodendroglia are sensitive to activation of the HPA axis and subsequent release of stress hormones as they express MRs and GRs (Jenkins et al., 2014). Prolonged exposure to glucocorticoids suppresses NG2-OPC proliferation, and synthetic glucocorticoids inhibit oligodendrocyte death. Stress hormones, or their analogs, can have protective effects when the exposure is acute. For example, treatment with methylprednisolone, a GR agonist, resulted in typical rates of cell death of cells expressing neuronal markers but preferential survival of cells expressing oligodendrocyte markers (Lee et al., 2008). Similarly, exposure to physiological (physical restraint) or pharmacological (CORT injection) stress resulted in a skewed ratio of neuron to glia in terms of neurogenic development (Chetty et al., 2014). For example, while under typical conditions, the newly born cells in the subgranular zone of the hippocampus develop primarily into neurons but following stress exposure and stress hormone exposure, there was a significant increase in the number of oligodendrocytes and a reduced number of neurons (Chetty et al., 2014). Additionally, exposure to dexamethasone on oligodendrocytes in culture results in increased rates of myelin formation and a subsequent increase in the overall quantity of myelin (Chan et al., 1998). However, excessive exposure to CORT has been shown to suppress proliferation of NG2-OPCs (Alonso, 2000; Wennstrom et al., 2006) and this effect does not appear to be age dependent, as decreases in both MBP expression and the number of oligodendrocytes are reported in fetal animals subjected to a GR agonist, betamethasone (Kumar et al., 2007).

These findings are of further importance as they shed light into the cellular pathology of human depression and affective disorders. Studies of chronically depressed human subjects have reported significant reductions in the number of glia in frontal cortical areas (Ongur et al., 1998; Rajkowska et al., 1999; Uranova et al., 2004; Rajkowska and Miguel-Hidalgo, 2007), findings which are paralleled in animal models of chronic stress. Rodents which were subject to chronic social defeat, a model of chronic stress (Rygula et al., 2005, 2006a,b; Krishnan et al., 2008), had a significantly depressed rate of cell proliferation in the mPFC, however, the percentage of the proliferating cells which were NG2-OPCs was comparable to non-stressed controls (Czeh et al., 2007). As human patients diagnosed with chronic depression are reported to have dysfunction of the HPA axis resulting in systemically elevated CORT levels (Stokes, 1995; Pariante and Miller, 2001; Burke et al., 2005; Cieslik et al., 2007), the reduced number of NG2-OPCs in these patients corresponds to the preclinical findings with chronic stress or artificial stress hormone administration. Further supporting this damaging relationship of stress hormones and NG2 proliferation is the work with antidepressant therapies, both pharmacological (i.e., fluoxetine) and physical (i.e, electroconvulsive therapy), which produced measureable increases in the number of NG2-OPCs (Kodama et al., 2004; Wennstrom et al., 2004; Czeh et al., 2007). Taken together, there is a clear link between exposure to chronic stress hormones and the dysregulation of NG2-glia proliferation and differentiation.

Oligodendroglia express dopaminergic receptors, and their activation can drive and influence development of premyelinating oligodendrocytes and myelin; the activation of D2 and D3 receptors on oligodendrocytes following oxidative stress injury can reduce glial loss. For example, NG2-OPCs have been shown to express dopamine receptors, specifically the D3 receptor (Bongarzone et al., 1998), and there is evidence that analogs of these glial precursors in human embryonic stem cell cultures express serotonergic receptors (5HT_2A_; Schaumburg et al., 2008). However, there has been no clear establishment of the function of dopamine or serotonergic receptors in NG2-OPCs with regard to disease pathology. However, a link has been established associating the diagnosis of mood disorders and NG2 proliferation, and while a likely biochemical mediator of these effects is the dysregulation of the monoamiergic system, it is not clear in which order the events occur; does deleterious behavior precede biochemistry implying NG2 proliferation as a pathologic response, or does a change in cortical function at a cellular level lead to pathological cortical function, implying that NG2 dysfunction is potentially driving the disease state? Future studies directed at specifically answering this question, as well as the precise involvement and function of monoaminergic receptors will be required to elucidate these processes.

## CONCLUDING THOUGHTS

In conclusion, there is substantial evidence that NG2-OPCs cells are critical for homeostatic control of oligodendrocytes in the adult brain. There is also evidence demonstrating that under pathologic conditions, NG2-OPCs can play a role in potential repair processes. Similarly, further studies are required in the future to confirm the neurogenic potential of NG2-OPCs particularly during adulthood, findings which could aid in the understanding of the role in NG2-OPCs in addiction. The prospective role of NG2-OPCs in the cortical pathology associated with drug addiction is a promising and underexplored area of research for investigating both pathophysiological mechanisms and potential strategies for recovery. NG2-OPCs are therefore a novel cell type requiring critical investigation into their function and role in the mammalian cortex.

## Conflict of Interest Statement

The authors declare that the research was conducted in the absence of any commercial or financial relationships that could be construed as a potential conflict of interest.
